# Antibody targeting of mutant calreticulin in myeloproliferative neoplasms

**DOI:** 10.1111/jcmm.17896

**Published:** 2023-08-07

**Authors:** Frederike Kramer, Ann Mullally

**Affiliations:** ^1^ Division of Hematology, Department of Medicine, Brigham and Women's Hospital Harvard Medical School Boston Massachusetts USA; ^2^ Department of Medical Oncology Dana‐Farber Cancer Institute Boston Massachusetts USA; ^3^ Broad Institute Cambridge Massachusetts USA

## Abstract

Mutations in calreticulin are one of the key disease‐initiating mutations in myeloproliferative neoplasms (MPN). In MPN, mutant calreticulin translates with a novel C‐terminus that leads to aberrant binding to the extracellular domain of the thrombopoietin receptor, MPL. This cell surface neoantigen has become an attractive target for immunological intervention. Here, we summarize recent advances in the development of mutant calreticulin targeting antibodies as a novel therapeutic approach in MPN.

## Graphical abstract

Overview of pathogenic mechanisms of mutant calreticulin (CALR) in myeloproliferative neoplasms (MPN) and potential ways to target the mutant calreticulin neoantigen by therapeutic monoclonal antibodies (mAb). 1: Mutant calreticulin is secreted and functions as an immunosuppressor. The mutant calreticulin novel C‐terminus is shown in red. 2: Aberrant binding of mutant calreticulin dimers to the thrombopoietin receptor MPL mediates receptor dimerization and leads to constitutive activation of JAK‐STAT signaling, promoting proliferation of MPN cells. Mutant calreticulin‐MPL binding occurs in the endoplasmic reticulum (ER), with the protein complex trafficking through the Golgi to the cell surface. 3: Therapeutic antibody targeting the novel C‐terminus of mutant calreticulin on the cell surface can prevent dimerization and activation of MPL (i.e. a blocking antibody). A mutant calreticulin antibody with an Fc component could be engineered to enable Fcγ receptor‐mediated immune cell targeting. 4: A bispecific approach targeting both MPL and mutant calreticulin could increase specificity and circumvent trapping of the mAb by secreted mutant calreticulin. 5: mAb‐toxin conjugates could increase efficacy. 6: The mutant calreticulin neoantigen could be targeted by engineered chimeric antigen receptor (CAR)‐T cells. 7: Bispecific antibodies could enable binding of cytotoxic CD3+ T‐cells to mutant calreticulin expressing MPN cells. 8: Circulating mutant calreticulin may bind the mAb and potentially act as a decoy, decreasing mAb availability. Figure was created using BioRender.

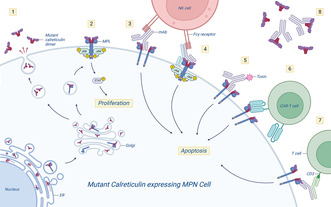

## 1 INTRODUCTION

Myeloproliferative neoplasms (MPN) are malignant haematological disorders that are caused by clonal proliferation of haematopoietic stem cells (HSC) in the bone marrow. There are three classical, BCR‐ABL‐negative MPN: essential thrombocythemia (ET), polycythemia vera (PV) and primary myelofibrosis (PMF), which present with distinct clinical features. Whereas ET and PV are characterized by platelet and erythrocyte overproduction, respectively, PMF is marked by aberrant proliferation of cells of the megakaryocytic lineage and progressive bone marrow fibrosis.^1^ All three classical, BCR‐ABL‐negative MPN are initiated by acquired, somatic mutations in HSC. In more than 90% of MPN patients, one of three driver mutations in *JAK2*, *CALR* or the thrombopoietin receptor *MPL* are present.^2^, ^3^, ^4^, ^5^, ^6^, ^7^, ^8^ Mutations in *CALR* are responsible for 20%–30% of MPN cases and, like the other driver mutations, lead to the constitutive activation of Janus kinase–signal transducer and activator of transcription (JAK–STAT) signalling.^9^

## 2 PATHOGENIC MECHANISMS OF MUTANT CALRETICULIN IN MPN

The majority of MPN driver mutations in *CALR* are caused by two mutations in exon 9 of the *CALR* gene, typically occurring in a heterozygous manner: (i) a 52 bp deletion (type I mutation), present in approximately 50% *CALR*‐mutant patients, and (ii) a 5 bp insertion (type II mutation), present in approximately 30% *CALR*‐mutant patients. The majority of the remaining *CALR* mutations are classified as type I‐like or type II‐like, with these categories defined on the basis of the deletion of stretches of negatively charged amino acids in the wild‐type calreticulin C‐terminus.^5^, ^6^ All MPN‐associated *CALR* mutations lead to a +1 bp frameshift that results in translation of an altered C‐terminus of the calreticulin protein. The novel C‐terminus is lacking the endoplasmic reticulum (ER) retention signal (KDEL domain) and primarily consists of positively charged amino acids. Since its discovery in 2013, the mechanisms by which mutant calreticulin causes MPN were elucidated by several groups. Mutant calreticulin forms homomultimers via its novel C‐terminus and acquires a pathogenic binding interaction with MPL in the ER.^10^ Lacking the KDEL domain, the mutant calreticulin‐MPL complex is shuttled to the cell surface.^11^, ^12^, ^13^ Mutant calreticulin stabilizes trans‐membranous MPL, resulting in ligand‐independent activation of MPL and downstream JAK–STAT signalling pathway activation.^11^, ^13^, ^14^, ^15^, ^16^, ^17^ Consequently, MPL expression is required for cell transformation by mutant calreticulin.^11^, ^12^, ^16^ For MPL binding, mutant calreticulin requires the positive electrostatic charge of the novel C‐terminus, as well as the lectin‐dependent function.^11^, ^13^, ^14^ Recent work has provided detailed insights into the protein conformation of mutant calreticulin and the formation of a tetrameric mutant calreticulin‐MPL complex, resulting in MPL dimerization and activation.^18^ Enhanced accessibility of the N‐terminal N‐glycan binding domain of mutant calreticulin facilitates binding to the extracellular domain of an immature, partially glycosylated MPL, while the mutant C‐terminus of mutant calreticulin also interacts with MPL via acidic patches (e.g. TFED, PDQEE and WEEP) in the extracellular domain of MPL.^18^ Cell intrinsically, mutant calreticulin promotes megakaryocytic differentiation through MPL activation, which is consistent with the fact that *CALR* mutations engender MPN with a megakaryocytic lineage phenotype (i.e. ET and PMF).^19^ Several groups have shown that wild‐type as well as mutant calreticulin are secreted, and become damage‐associated molecular patterns exhibiting immunomodulatory functions.^20^, ^21^ Whereas secretion of wild‐type calreticulin is mainly considered to be a reaction to ER stress,^22^ other studies indicate that in MPN, secretion of mutant calreticulin is facilitated by the lack of the KDEL domain. Interestingly, the majority of soluble mutant calreticulin detectable in the plasma has been found to be secreted from non‐MPL‐expressing cells in MPN.^23^ Circulating mutant calreticulin has been shown to have an immunosuppressive role, for example, by reducing phagocytosis mediated by CD11c^+^ bone marrow‐derived dendritic cells.^24^

## 3 CURRENT TREATMENT OPTIONS FOR *CALR* ‐MUTANT MPN

Treatment of MPN relies on cytoreductive agents, including hydroxyurea, pegylated interferons, as well as the JAK1/2 inhibitor, ruxolitinib. Cytoreductive therapy can reduce blood counts, thrombotic risk, splenomegaly and improve symptoms.^25^, ^26^ Despite these benefits, none of the current medical treatments for MPN eliminate the disease‐initiating *CALR*‐mutant HSC clone. Over time, patients develop resistance to JAK2 inhibition, further limiting the efficacy of ruxolitinib. To date, the only curative treatment for *CALR*‐mutant MPN remains allogeneic stem cell transplantation, a procedure associated with substantial morbidity, in addition to a mortality risk. With the presentation of a neoantigen, the development of therapeutic antibodies targeting the novel cell surface mutant calreticulin C‐terminus has become a strategy of great interest for the inhibition of pathogenic MPL activation.

## 4 TARGETED THERAPY USING MUTANT CALRETICULIN TARGETING MONOCLONAL ANTIBODIES

The development of therapeutic, monoclonal antibodies (mAbs) for cancer therapy has been successful, broadly speaking (e.g. targeting CD20 in non‐Hodgkin lymphoma). The progress in unravelling the structural properties and the mechanisms of the pathogenic interaction of mutant calreticulin with MPL provides the understanding needed to translate this knowledge into efficacious treatment. Recent research efforts have therefore centered on immunotherapeutic approaches with the goal of targeting the mutant calreticulin neoepitope while sparing normal haematopoiesis. In 2020, Kihara et al. reported (in abstract form) the generation of the mouse chimeric monoclonal antibody B3, specifically targeting mutant calreticulin.^27^ In a *CALR* ^del52^ ET mouse model, treatment with B3 reduced platelets in the peripheral blood and numbers of megakaryocytes in the bone marrow of the mice. Soon after, Achyutuni and colleagues generated a murine IgG2a raised against the human calreticulin neoantigen and treated homozygous *CALR* ^del52^ transgenic mice.^28^ Although treatment with the antibody was only for 2.5 days (5 doses total), the platelet count rapidly dropped before rising again 24 h after completion of treatment.^28^ In 2022, Mughal et al. generated and characterized eight peptide antibodies recognizing mutant calreticulin. This study provides important information on essential sites within mutant calreticulin epitopes, however these antibodies have yet to be tested in pre‐clinical models.^29^ In the same year, Tvorogov et al. reported the development of the monoclonal antibody 4D7, targeting mutant calreticulin on the cell surface.^30^ 4D7 was generated using a synthetic peptide corresponding to the novel C‐terminus of mutant calreticulin using a hybridoma approach. The antibody is directed against the common C‐terminus of both Type I and Type II *CALR* mutations, and it effectively blocked binding of mutant calreticulin to MPL, abrogating aberrant JAK–STAT activation. Tvorogov and colleagues also showed that 4D7 inhibited TPO‐independent megakaryocyte differentiation in patient‐derived *CALR*‐mutant CD34^+^ cells. Treatment with 4D7 did not show any inhibitory effect on in vitro haematopoiesis in non‐mutated cells. The authors further showed that 4D7 has efficacy on ruxolitinib resistant cells in vitro, suggesting that treatment with 4D7 might be a promising therapeutic approach for patients with an acquired ruxolitinib resistance. 4D7 showed beneficial effects on survival in cell line xenograft models, both in calreticulin‐mutant as well as ruxolitinib‐resistant cells. The antibody has yet to be tested in patient‐derived xenograft models or in genetic MPN mouse models. Another milestone in the development of mutant calreticulin‐targeting antibodies was the generation of the human IgG1 mAb INCA033989, introduced by Reis et al. in a plenary abstract at the 2022 American Society of Haematology (ASH) meeting.^31^ Based on the data presented in the abstract (currently unpublished), INCA033989 inhibited mutant calreticulin‐induced MPL signalling in murine Ba/F3 cells, but showed no effects on non‐mutated cells. Reis et al. also reported enhanced efficacy of INCA033989‐ruxolitinib combination in mouse cells in vitro. In patient‐derived CD34^+^ cells, treatment with INCA033989 inhibited JAK–STAT signalling and proliferation of progenitor cells, an effect not observed in non‐mutated or *JAK2*‐mutant cells. In competitive transplant calreticulin‐mutant mouse models, 10 weeks of treatment with INCA033989 prevented thrombocytosis and significantly decreased the numbers of *CALR*‐mutant stem and progenitor cells, as well as megakaryocytes in the bone marrow, without affecting cellularity of wild‐type mice. Secondary transplantation did not result in development of MPN in mice, suggesting that treatment with INCA033989 successfully targeted the *CALR‐*mutant MPN disease‐propagating HSC. The expectation is that INCA033989 will enter Phase 1 clinical trials in patients with *CALR*‐mutant MPN in 2023 [verbal communication, Dr. Reis, ASH plenary presentation 2022].

## 5 FUTURE DIRECTIONS

Research in the last few years has shown immense progress in developing a specific, mutant calreticulin targeting treatment approach in MPN. Of note, other promising immunological approaches, such as mutant calreticulin peptide vaccination and T cell‐directed targeting have been investigated and reviewed elsewhere,^32^, ^33^ and are therefore not the primary focus of this review. To date, peptide vaccination targeting the mutant calreticulin neoantigen is the immune therapy approach that has advanced the furthest clinically. One Phase 1 vaccine trial (NCT03566446), using a 36 amino acid peptide vaccine spanning the novel mutant calreticulin C terminus, has been completed and the vaccine was found to be safe and tolerable.^34^ While 8/10 patients with MPN who received the peptide vaccine showed evidence of T‐cell responses, no patient demonstrated a clinical response.^34^ There are several potential reasons why a mutant calreticulin‐directed peptide vaccine might not induce an immune response in patients, including (i) the patients who received the vaccine may not have expressed human leukocyte antigen (HLA) subtypes that present the mutant calreticulin neo‐epitope with high affinity, (ii) defects in major histocompatibility complex (MHC)‐mediated presentation of the mutant calreticulin neo‐epitope, (iii) inadequate immune stimulation by the adjuvant and (iv) an immunosuppressive microenvironment in the context of MPN‐related chronic inflammation. Two other mutant calreticulin vaccine trials (NCT05444530 and NCT05025488) are currently open, with some differences in their approaches compared to the published trial and results from these ongoing studies are eagerly awaited.

Targeting mutant calreticulin with a cell‐surface blocking mAb may circumvent some of the challenges of peptide vaccination. Recent advances in the development of mutant calreticulin targeting mAb have taken advantage of the blocking properties of the antibody binding to the cell surface neoantigen, preventing MPL dimerization, activation and thus abrogating aberrant activation of JAK–STAT signalling. INCA033989, which has been tested in pre‐clinical models by Reis et al., showed efficacy as an Fc‐silent IgG1, and the abrogation of JAK–STAT signalling indicates successful inhibition of MPL activation. Based on its mechanism of action, INCA033989 is expected to inhibit proliferation of *CALR*‐mutant cells, however it remains to be determined if treatment with INCA033989 will preferentially target the mutant *CALR* clone in patients to achieve molecular responses and/or remissions. While the lack of the Fc domain reduces toxicity, it also precludes Fc‐mediated cell death that could improve therapeutic potency of the antibody. Future efforts could therefore be aimed at optimizing treatment efficacy by Fc engineering to enable Fcγ receptor‐mediated antibody‐dependent cellular cytotoxicity or phagocytosis. Additional approaches to enhance the efficacy of the mutant calreticulin mAb may include the development of antibody‐drug conjugates, and/or integrating the antibody into a chimeric antigen receptor (CAR)‐T construct. CAR T therapy, a significant advance in oncology immunotherapy, has been successfully used in ALL and lymphoma.^35^, ^36^ A constraint of targeting mutant calreticulin using therapeutic antibodies could be circulating mutant calreticulin, potentially acting as decoy for antibody binding, thus reducing the availability of the mutant calreticulin mAb to bind on the cell surface. Even though it might be advantageous to prevent secreted mutant calreticulin from (i) binding extracellularly to the mutant calreticulin‐MPL complex on the cell surface and (ii) mediating its cell‐extrinsic effects as an immunosuppressor, the potential therapeutic benefit of blocking secreted mutant calreticulin is currently unclear. Developing a bispecific antibody, targeting both mutant calreticulin and MPL in the complex, could enhance specificity by selectively targeting the protein complex, and preventing secreted mutant calreticulin from functioning as a decoy. A bispecific approach could also be applied to allow concomitant binding of mutant calreticulin expressing MPN cells and T cells. This approach has been successfully used to target CD19 in acute lymphoblastic leukaemia (ALL) through engaging CD3 on T‐cells.^37^

## 6 CONCLUSION

MPN are a group of chronic blood cancers that have an unmet need for treatment options which eliminate the disease‐propagating clone. Recent advances in identifying the mechanisms by which mutant calreticulin causes MPN paved the path for immunological targeting of *CALR*‐mutant MPN cells, and specific mutant calreticulin targeting mAbs have been developed and found to be efficacious in preclinical mouse models. The safety and efficacy of these novel antibodies have yet to be evaluated in MPN patients.

## AUTHOR CONTRIBUTIONS

**Frederike Kramer:** Conceptualization (lead); writing – original draft (lead). **Ann Mullally:** Funding acquisition (lead); supervision (lead); writing – review and editing (lead).

## CONFLICT OF INTEREST STATEMENT

AM receives research funding from Morphic and Relay. A.M. has consulted for Janssen, PharmaEssentia, Constellation, Aclaris, Cellarity, Morphic, Biomarin, Protagonist and Incyte. AM has received research funding from Janssen and Actuate Therapeutics and a speaker honorarium from AOP Health.

## Data Availability

Data sharing not applicable ‐ no new data generated, or the article describes entirely theoretical research.
